# Increasing the Use of Skilled Health Personnel Where Traditional Birth Attendants Were Providers of Childbirth Care: A Systematic Review

**DOI:** 10.1371/journal.pone.0047946

**Published:** 2012-10-24

**Authors:** Claudia Vieira, Anayda Portela, Tina Miller, Ernestina Coast, Tiziana Leone, Cicely Marston

**Affiliations:** 1 Independent consultant, Lisbon, Portugal; 2 Department of Making Pregnancy Safer, World Health Organization, Geneva, Switzerland; 3 Department of Maternal, Newborn, Child and Adolescent Health, World Health Organization, Geneva, Switzerland; 4 Department of Social Sciences, Oxford Brookes University, Oxford, United Kingdom; 5 Department of Social Policy, London School of Economics, London, United Kingdom; 6 Faculty of Public Health and Policy, London School of Hygiene and Tropical Medicine, London, United Kingdom; University of Hong Kong, China

## Abstract

**Background:**

Improved access to skilled health personnel for childbirth is a priority strategy to improve maternal health. This study investigates interventions to achieve this where traditional birth attendants were providers of childbirth care and asks what has been done and what has worked?

**Methods and Findings:**

We systematically reviewed published and unpublished literature, searching 26 databases and contacting experts to find relevant studies. We included references from all time periods and locations. 132 items from 41 countries met our inclusion criteria and are included in an inventory; six were intervention evaluations of high or moderate quality which we further analysed. Four studies report on interventions to deploy midwives closer to communities: two studies in Indonesia reported an increase in use of skilled health personnel; another Indonesian study showed increased uptake of caesarean sections as midwives per population increased; one study in Bangladesh reported decreased risk of maternal death. Two studies report on interventions to address financial barriers: one in Bangladesh reported an increase in use of skilled health personnel where financial barriers for users were addressed and incentives were given to skilled care providers; another in Peru reported that use of emergency obstetric care increased by subsidies for preventive and maternity care, but not by improved quality of care.

**Conclusions:**

The interventions had positive outcomes for relevant maternal health indicators. However, three of the studies evaluate the village midwife programme in Indonesia, which limits the generalizability of conclusions. Most studies report on a main intervention, despite other activities, such as community mobilization or partnerships with traditional birth attendants. Many authors note that multiple factors including distance, transport, family preferences/support also need to be addressed. Case studies of interventions in the inventory illustrate how different countries attempted to address these complexities. Few high quality studies that measure effectiveness of interventions exist.

## Introduction

Maternal deaths remain unacceptably high in many parts of the world. Some progress has been made: the proportion of births attended by skilled health personnel increased in developing regions from 55% to 65% between 1990 and 2009 [1], and worldwide the maternal mortality ratio has fallen since 1990– probably related to improved access to skilled care and to antenatal care [2]. Nevertheless, in 2010, approximately 287,000 women died in childbirth or from pregnancy complications, most of them in poorer countries [2]. Better access to skilled health personnel for childbirth is a priority strategy and a key indicator for Millennium Development Goal (MDG) 5a to improve maternal health.

Where skilled health personnel and institutional facilities for childbirth are absent, one strategy to improve access has been to train traditional birth attendants (TBAs) [3]. In the 1970s, international organizations, including the World Health Organization (WHO), invested in TBA training. Training TBAs for childbirth care, however, has been found not to reduce maternal mortality [4]–[6], and by 1997, attention turned towards “skilled birth attendants” [7]. A “skilled attendant” was defined in a joint statement issued by the WHO, the International Confederation of Midwives and the International Federation of Gynaecology and Obstetrics in 2004 [8]. The statement identified the skills and abilities required for a skilled attendant, and does not include TBAs, although countries are encouraged to work with TBAs to define new roles for them, and ensure good working relations between TBAs, skilled attendants, and staff in referral facilities. In this paper, in line with current terminology, we use the term skilled health personnel.

There is no global estimate of the number of births attended by TBAs. Between 130 and 180 million births will not be attended by skilled health personnel in South Asia and sub-Saharan Africa between 2011 and 2015, with 90% of these births in rural areas [9]: many will be attended by TBAs. TBAs also operate in urban areas, where they can be an important source of childbirth care for the poorest populations [10].

Even with the 2015 deadline to meet MDG 5 looming, we still do not know enough about what interventions might increase births with skilled health personnel where TBAs are providers of care. A recent systematic review examined integration of TBAs with formal health systems and found that integration can increase use of skilled birth attendants [11]. The review included efforts where TBAs are trained to continue childbirth care. Our review examines efforts to move away from TBAs as providers of childbirth care and asks the wider question: what has worked to increase use of skilled health personnel where TBAs were providers of childbirth care? We systematically review the literature to examine the interventions undertaken and their effects on the use of skilled health personnel by women for childbirth care. In addition to identifying high-quality evidence that addresses this question, we have compiled an inventory of references which describe the different interventions undertaken in different countries.

## Methods

**Figure 1:**
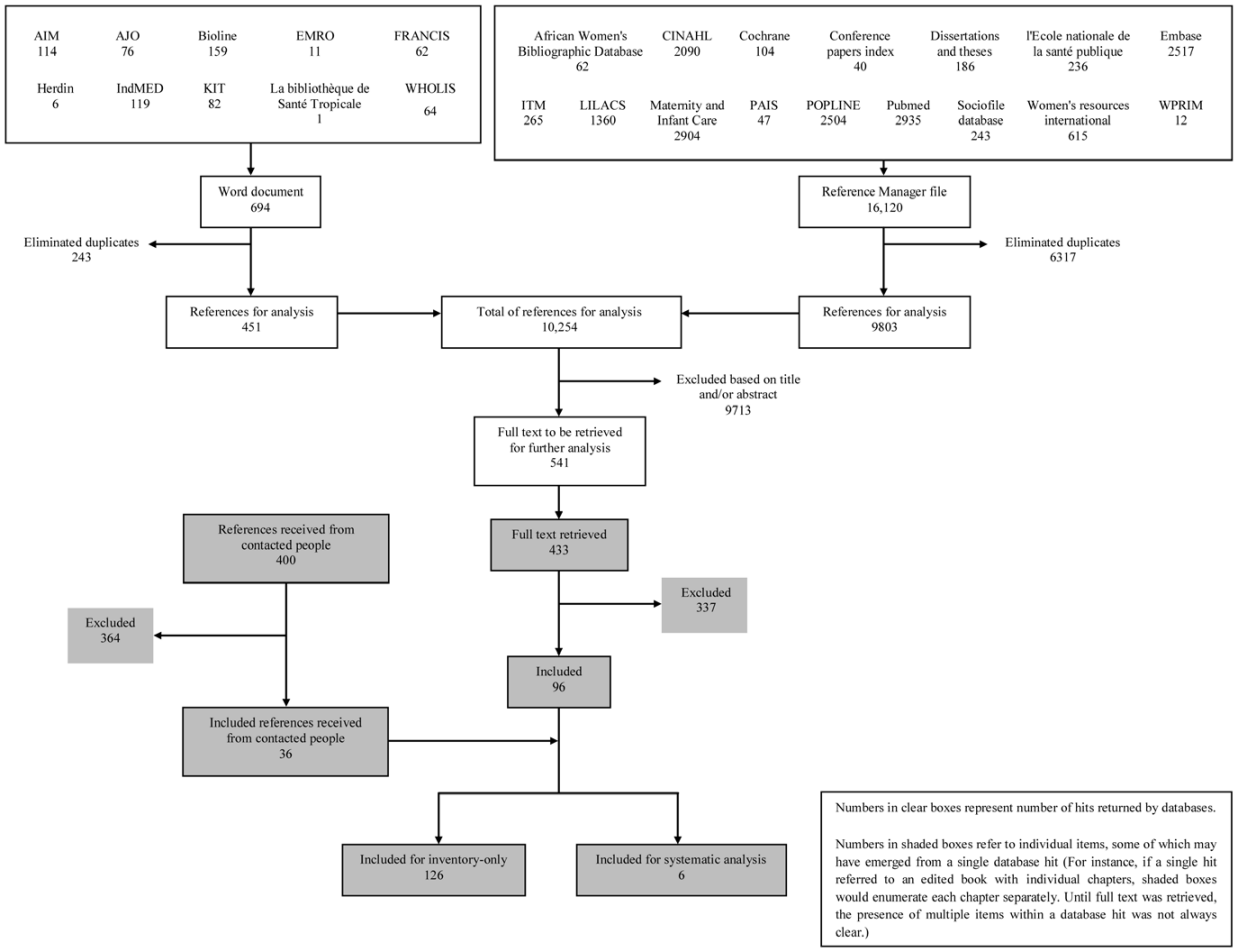
Flow diagram of the systematic search process and inclusion of references for the systematic review (modified from the PRISMA flow diagram). Some databases did not allow for a direct importation of references to Reference Manager and were thus managed using Microsoft Word. Abbreviations: AIM: African Index Medicus; AJO: African Journal Online; CINAHL: Cumulative Index to Nursing and Allied Health Literature; EMRO: Eastern Mediterranean Regional Office; Herdin: Health Research and Development Information Network; ITM: Institute of Tropical Medicine in Antwerp Belgium; LILACS: Latin American and Caribbean Health Sciences; POPLINE: Population Information Online; KIT: Royal Tropical Institute; WPRIM: Western Pacific Region Index Medicus; WHOLIS: World Health Organization library database.

Following the Preferred Reporting Items for Systematic Reviews and Meta-Analyses (PRISMA) statement for the transparent reporting of systematic reviews [12], we finalized our protocol after comment and review by 11 experts (Protocol S1). We performed a preliminary search to identify appropriate databases, querying 87 databases between 18 February and 10 March 2010 using: “traditional birth attendants” (for English indexed databases) and “accoucheuses traditionnelles” (for French indexed databases). We selected 26 databases (Figure 1) and conducted the full search between 10 May and 17 June 2010 using the strategy shown in Box 1. We requested published and unpublished materials from agencies, discussion forums and experts in the field. Papers were received until January 2012.

**Figure:**
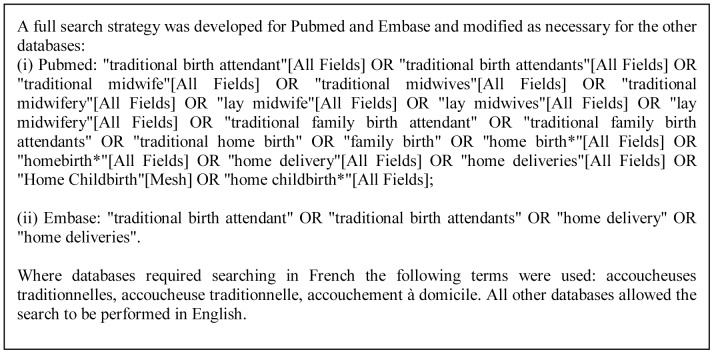
Box 1. Search strategy.

### Inclusion and Exclusion Criteria

We sought literature from all time periods and any location, describing interventions to increase birth with skilled health personnel, in settings where TBAs were providers of childbirth care. We included references in English, French, Spanish or Portuguese where:

TBAs had been attending births prior to the intervention; and
a transition to skilled health personnel was in progress or planned.

We excluded references where:

there was no specific mention of TBAs attending births;
TBAs continued to attend births and referred women to skilled health personnel or a facility for complications;
the intervention was to train TBAs to upgrade their skills to attend birth, or where they were supplied with delivery kits to continue to provide childbirth care; and
barriers to use of care with skilled health personnel or women’s preferences for care were described but no intervention examined;

Study design was not an exclusion criterion at this stage.

We then employed two quality assessment tools designed for quantitative [13] and qualitative studies [14]. For mixed methods studies, both tools were applied. We designated studies as high, moderate or low quality, defined as follows: High - quality of the research design and findings reported allow confidence in measures of internal and external validity; Moderate - quality of the research design and findings reported in the publication correspond to some quality assessment criteria, allowing for some level of confidence in the outcomes described, additional information on aspects of the research process would increase confidence; and Low - quality of the research design and findings correspond to few quality assessment criteria, and lack details that would allow confidence in the outcomes described.

We used a pre-designed data recording table for data extraction, based on the criteria proposed by the Strengthening the Reporting of Observational Studies in Epidemiology (STROBE) statement [15], the Transparent Reporting of Evaluations with Non-randomized Designs (TREND) statement [16] and the National Public Health Partnership [17].

CV conducted electronic searches, retrieved the full text, contacted experts, agencies and authors and reviewed all retrieved abstracts for eligibility. AP independently reviewed 50% of the retrieved abstracts. Full references were retrieved where necessary to determine eligibility. Differences were resolved by discussion. AP, CM, TM and EC independently assessed studies for quality. We agreed quality designation by consensus. All authors extracted data from the selected papers. We retained moderate and high quality intervention studies for analysis. All authors participated in the analysis and the final write-up of the study.

## Results

The electronic searches yielded 16,814 references from 26 databases (Figure 1), which we managed with Reference Manager and Microsoft Word. 10,254 references remained after elimination of duplicates; 541 after exclusion based on the title and/or abstract. We retrieved/received 433 full texts via libraries, web searches, and direct requests to authors and 400 references from agencies and experts in the field.

132 references fitted our inclusion criteria (Table S1). These are all documented because they provide information on interventions that have been implemented, but the majority (n = 97) either omit information about effectiveness on outcomes (increased utilization of skilled health personnel services or improved maternal and newborn health outcomes) or were not designed to capture such data. The remaining 35 studies which contained effectiveness information were put forward for quality appraisal. Of these, 12 were “too complex” for inclusion: they covered extended time periods with multiple and changing interventions where it was not possible to attribute reported outcomes to specific interventions.

After removing the 12 “too complex” studies (see above), and the low quality studies (n = 17), six high or moderate quality studies remained (Table 1). These six studies varied in design, use of control groups, data collection methods, analysis, and reporting of findings. None used randomized controlled trials. All six contained clear descriptions of research design, methods and data analysis, and demonstrated a critical approach, e.g. reflecting on possible sources of bias. They used at least one comparison group, but intervention and comparison groups were not usually randomly assigned. For example, assessment of the Indonesian village midwife programme [18], [19], used comparison groups retrospectively determined using longitudinal datasets, where midwives had yet not been assigned, or where they were never assigned. We judged that even comparison groups with limitations helped indicate to some extent whether or not any observed changes could be ascribed to an intervention.

**Table 1:** Studies included in the systematic review.

Reference	Country	Study aims	Design	Context/intervention description	Outcomes/results
Shrestha R (2007) Family planning, community health interventions and the mortality risk of children in Indonesia: PhD thesis. The Ohio State University. 108 p. [unpublished work].	Indonesia	To assess the impact of the village midwife programme on infant mortalityand women’sutilization ofantenatal andchildbirth services.	Retrospective pregnancy history data (where available) was taken from three waves of the longitudinal Indonesian Family Life Survey (IFLS, 1993, 1997 and 2000). Interview data was also collected from IFLS households and key community informants. Areas where the midwife programme was introduced provided the intervention sites.	Government-funded village midwife programme started in 1989 that trained and placed over 54,000 midwives in villages across Indonesia, to provide antenatal, intrapartum and postpartum care to village women. The programme promoted community participation in health, undertook work with traditional birth attendants (TBAs) and referred complicated cases to health centres and hospitals. Following initial government funding midwives were expected to become funded through private practice. (See also Frankenberg et al., 2009 and Achadi et al., 2007, this table)	Place of birth and skilled assistance at birth changed as a result of the village midwife programme. Births in a hospital, midwifery or physician’s office or village delivery post increased and there was a decrease in births in the home. Intervention areas showed an increase from 15.9% in 1988 to 40.4% in 1999 of births in a hospital, midwifery or physician’s office or village delivery post; while home births decreased from 79.5% to 58.1% in the same period.Births with a midwife increased from 36.9% to 55.0% between 1988 and 1999 in the full sample and from 25.3% to 51.9% in the intervention areas. Births with a physician also increased from 5.5% to 12.6% for the same period in the full sample and from 0.0% to 8.6% in the intervention areas.
Frankenberg E, Buttenheim A, Sikoki B, Suriastini W (2009) Do women increase their use of reproductive health care when it becomes more available? Evidence from Indonesia. Stud Fam Plann 40∶27–38.	Indonesia	To assess the impact of the village midwife programme andits expansion ofmidwifery services on women’s utilization of antenatal and childbirth services.	A quasi experiment which used data from three rounds of the IFLS, (1993, 1997, 2000) to compare utilization of antenatal and childbirth services over sequential pregnancies. The samples comprised women in intervention communities (where a village midwife had been placed) and control communities (no village midwife placed). A fixed effect model was used to contrast service utilization behaviours between the two communities.	The government-funded village midwife programme (*bidan di desa*) began in 1989 and trained and placed over 54,000 midwives in villages across Indonesia, to provide antenatal, intrapartum and postpartum care to village women, placing midwives closer to women. The programme promoted community participation in health, undertook work with TBAs and referred complicated cases to health centres and hospitals. Following initial government funding midwives were expected to become funded through private practice. (See also Shrestha, 2007 and Achadi et al., 2007, this table.)	The presence of midwives was significantly positively associated with measures of antenatal care (uptake of antenatal care, receipt of care in first trimester, receipt of iron tablets during pregnancy). However, presence of midwives was negatively associated with “medically-oriented delivery care”. High reliance on TBAs in the intervention areas could be continuing because of greater traditionalism in those villages when compared to the control areas. When they included a mother-specific fixed effect, women in this model were about 1.7 times more likely to have a “medically-oriented delivery” where midwives were available than where they were not.
Achadi E, Scott S, Pambudi ES, Makowiecka K, Marshall T, et al. (2007) Midwifery provision and uptake of maternity care in Indonesia. Trop Med Int Health 12∶1490–1497.	Indonesia	To examine the association between midwife density, geographical location, numberof births attendedby a healthprofessional, andbirths withcaesarean section.The study wasconducted in twodistricts ofIndonesia Serang(moderately urban)and Pandegang(a more remotearea).	Four sources of data were used: i) a census of midwifery providers (2005); ii) a stratified cluster, random sample survey of women with a live or stillbirth in the last 2 years. Survey conducted between April and June 2006;iii) a census of all caesarean births in the four hospitals serving the two study districts. This data was collected retrospectively from admissions between November 2003 and October 2004; iv) data from the National Statistics Office on size and population of each village and distance from a hospital.	Government-funded village midwife programme (*bidan di desa*) began in 1989 and trained and placed over 54,000 midwives in villages across Indonesia, to provide antenatal, intrapartum and postpartum care to village women, placing midwives closer to women. By 1996, 96.2% of villages had a midwife.This midwife was either village-based, i.e., responsible for midwifery provision in one or more village, or health-centre based (but also may have been assigned responsibility for villages). (See also Frankenberg et al., 2009 and Shrestha, 2007, this table)	Birth with a health professional increased with increasing midwife density. However after adjusting for risk factors including midwife, village and individual woman characteristics, the association of birth with a health professional and midwife density disappeared. But uptake of caesarean sections significantly increased with the increase of midwife density. For both these outcomes proximity to the hospital was a significant factor (i.e., the closer to the hospital, the higher the uptake).Adjusted analysis also showed that increased levels of births with a health professional were associated with length of placement (five years) of a midwife in an assigned village (adjusted odds ratio (OR) 2.95 for ≥5, 95% CI 1.26, 6.90), whereas lower levels were associated with a higher number of TBAs.Women living in a village with a health centre were 1.67 times more likely to deliver by caesarean section (95% CI: 1.36, 2.06).
Fauveau V, Stewart K, Khan SA, Chakraborty J (1991) Effect on mortality of community-based maternity-care programme in rural Bangladesh. Lancet 338∶1183–1186.	Bangladesh	To evaluate a maternity-care programme to reduce maternal mortality, in the context of a community-based maternal and child health and family planning (MCH-FP) project.	Use of death forms and medical reports to compare “obstetric maternal mortality ratios” in a programme and neighbouring control site during the three years before and after the start of the programme. Ratio of deaths per thousand live births was used to measure the risk of dying during pregnancy.	Matlab, an area with a population of approximately 196,000, was divided into a treatment area and comparison area in 1977. The treatment area was where the MCH-FP project was implemented and the comparison site (of equal size) was served only by Government health services. In the MCH-FP project area the village community health workers (CHWs) were the principal providers of services which included a variety of health education and counselling services as well as detection and referral for mothers and children and distribution of safe delivery kits. In order to have a control area, the Matlab maternity-care programme was implemented in half of the MCH-FP project area (about 48,000 people in 39 villages). There were two health outposts in each programme and control area. “Two nurse-midwives (government trained) were recruited and posted in each outpost of the programme area.” (p. 1184). Their roles included working with CHWs and TBAs to ensure that CHWs (not TBAs) called them during labour; antenatal visits to women identified by CHWs; assessment of antenatal complications; attendance at as many home births as possible; treatment of complications before they became too severe; referral and accompanying woman to the central clinic at Matlab; visiting new mothers within 48 hours of birth.The programme also provided a referral chain, accompanying pregnant women to the maternity clinic at Matlab. Transfer by ambulance to the district hospital for caesarean was also implemented.	While direct “obstetric mortality ratios” in the pre-programme period (1984–86) were similar in the treatment and control areas (3.9 in the control area and 4.4 in the intervention area OR 1.14, (95% CI 0.59–2.21)), “obstetric mortality ratios” during the programme period (1987–89) were significantly lower in the intervention area (1.4) than the control area (3.8). OR 0.35, (95% CI 0.13–0.93).There was a significant reduction in maternal deaths in the intervention area over time (OR 0.31, (95% CI 0.11–0.81), but no such reduction in the control area.
Hatt L, Nguyen H, Sloan N, Miner S, Magvanjav O, et al. (2010) Economic evaluation of demand-aide financing (DSF) for maternal health in Bangladesh. Review, analysis and assessment of issues related to health care financing and health economics in Bangladesh. Bethesda: Abt Associates Inc. 152 p. [unpublished work].	Bangladesh	To describe and assess the operations and impact of a demand-side financing (DSF) maternal health voucher pilot programme.	Evaluation was conducted (June–Dec 2009) in the 21 DSF *upazilas* (sub-districts) where the programme was in place for at least two years. DSF intervention *upazilas* were compared with control *upazila*s, matched for various measures. Household survey also undertaken with women who had given birth in the previous 6 months in 16 of the 21 intervention *upazilas* (8 universal and 8 means-tested) and their matched controls. Qualitative data collected in 8 intervention *upazilas* using key informant interviews; provider interviews; facility quality assessments (also conducted in 8 matched control *upazilas*), and focus group discussions with women who had given birth in the previous 6 months.	In July 2004 the Ministry of Health and Family Welfare launched a pilot maternal health voucher programme in 2 *upazilas* to increase the use of qualified birth attendants and reduce the financial costs of childbirth. Vouchers were distributed to eligible pregnant women entitling them to free access to: 3 antenatal care (ANC) visits; childbirth with a qualified provider in a health facility or at home; emergency care for obstetric complications (including caesarean sections); 1 postnatal check-up; cash stipends for routine and emergency referral transportation; cash and in-kind incentives (a gift-box) for birth with a qualified provider. Programme incentives were also given to health care providers including private and non-governmental organization providers who identified eligible women and provided maternal health services.When first implemented in 2006, target beneficiaries in the 24 “means-tested” DSF *upazilas* were women who met stringent poverty-measure eligibility criteria (p. 8), but these criteria were relaxed in 2007 in 9 “universal” intervention *upazilas* entitling all permanent resident, pregnant women of parity 1 or 2, regardless of poverty status, to become beneficiaries of the scheme.At the time of the study the programme covered approximately 10.36 million people.	Demand side bivariate analysis:- More women in the universal and means-tested areas had at least one ANC visit during their last pregnancy (91.8% and 91.2% respectively) than in the control areas (76%) (p<0.001).- More women in the universal and means-tested areas gave birth with qualified providers (58.1% and 70.2%, respectively) than in the control areas (27.1%) (p<0.001).- More women in the universal and means-tested areas had a complicated birth attended by qualified providers (87.0% and 82.4% respectively) than in the control areas (47.8%) (p<0.001).- More women in the universal and means-tested areas gave birth in a health facility (43.9% and 30.1% respectively) than women in the control areas (18.7%) (p<0.001).The multivariate analysis confirmed the result that the DSF programme had a positive effect on the utilisation of maternal health services.Sub-analysis for the voucher recipients only reported that:- More voucher recipients in the universal areas had an institutional birth (52.5%) than those in the means-tested area (32.9%) (p = 0.009).- More voucher recipients in the means-tested areas had a home birth with a qualified provider (50.5%) than those in the universal areas (20.5%) (p = 0.009). However more voucher recipients in the universal areas had a home birth with an unqualified provider (27.0%) than those in the means-tested areas (16.6%) (p = 0.009).- More home births were attended by a community skilled birth attendant in the means-tested areas (54.3%) than in the universal areas (25.8%) and control areas (3.1%) (p<0.001). However more women in the control areas had home births attended by a doctor, nurse, midwife, paramedic or family welfare visitor (7.0%) than the universal or means-tested areas (1.4% and 3.4% respectively) (p<0.001)- More women had home births attended by unqualified attendants in the control areas (90.0%) than in the universal or means-tested areas (72.8% and 42.3% respectively (p<0.001).Supply side bivariate analysis:The average number of ANC visits during 2008 was significantly higher in voucher facilities than in control facilities.The incidence of c-sections was higher in the intervention areas (12.1%) than in the control facilities (4.4%) (p<0.001), while complicated births were lower in the intervention area (24%) compared to the control area (30%) (p<0.001).The incidence of complicated births was lower in intervention area (24%) than in the control facilities (30%) (p<0.001).
McQuestion MJ, Velasquez A (2006) Evaluating program effects on institutional delivery in Peru. Health Policy 77∶221–232.	Peru	To evaluate the effects of two Peruvian health programmes and women’s utilization of a public emergency obstetric care (EmOC) facility.	Phase I: midterm evaluation in 2000 of 37 facilities selected from a random sample of *Proyecto 2000* treatment facilities judged as high quality. Comparison group comprised 37 comparable facilities which had only received routine Ministry of Health supervision over the period. Phase II: 2002 second round of data collection undertaken. Treatment group comprised all 19 Phase I hospitals and a subset of 12 Phase I health centres. New control group comprised 15 of the Phase I control establishments and 14 additional establishments (all controls purposively selected to be similar).Facility data supplemented with household survey in treatment and control areas to assess changes in local utilization patterns and measure the national Maternal and Child Health Insurance (*SMI*) programme participation. The survey instrument included selected items from Peru’s DHS III and DHS IV e.g. household characteristics, birth histories and pregnancy-related behaviours.	In 1996 the Ministry of Health implemented *Proyecto 2000* in 12 *departmentos* with the highest maternal and neonatal mortality levels. From 1996–2002, the project aimed to improve the quality of EmOC (by also making services culturally acceptable) and increase use of public EmOC facilities via mass media, health education and social mobilization efforts.From 1998, the national *SMI* programme provided subsidized preventive and maternal care for eligible low-income pregnant women (including institutional childbirth in public EmOC facilities), mothers, and children aged 0–4 years. In 2001 the programme was implemented nationally, however many eligible households were not enrolled.	*Proyecto 2000* improved the quality of care in EmOC facilities but did not directly increase the probability of childbirth in a Ministry of Health EmOC facility.Five different behavioural models were analysed. Only reducing out-of-pocket costs via the *SMI* programme was associated with an increase in use of EmOC.

Three studies in Indonesia [18]–[20] and one study in Bangladesh [21] focused on interventions to deploy midwives closer to communities. The three Indonesian studies considered the impact of the village midwife programme (*bidan di desa*), which began in 1989. More than 54,000 midwives were trained and were either village-based (responsible for midwifery provision in one or more villages) or health-centre based (primary place of work was at the health centre, but who also may have been assigned responsibility for villages). As well as helping with childbirth, the midwives worked with TBAs, referred complicated cases to health centres and hospitals and promoted community participation in health.

Two of the studies in Indonesia [18], [19] used the longitudinal Indonesian Family Life Survey (IFLS) to evaluate the village midwife programme. One [18] used retrospective pregnancy histories from three waves of the IFLS (1993, 1997, and 2000) and identified a decline in both the use of TBAs at birth and the proportion of home births, alongside an increase in births with a midwife or skilled health personnel or in a facility. The improvements occurred across the full sample, but were significantly better in the intervention areas. An increase in coverage of the programme was also associated with a significant decline in neonatal mortality, although not post-neonatal mortality.

A separate study [19] of this programme, also using the IFLS data, examined the relationship between increased access to midwifery services and changes in use of antenatal and childbirth care. It concluded that there were differences in use of health care during pregnancy and childbirth in communities with village midwives, compared with those without. Women in programme areas were more likely to receive antenatal care and to give birth in a facility or with skilled health personnel. They also concluded that “access to village midwives has a stronger effect on receipt of antenatal care among women with relatively low levels of education than among their better-educated counterparts” (p. 34).

The third study - also reporting on the village midwife programme - in two districts of West Java, Indonesia [20] examined the relationship of uptake of maternity care, with midwife density - the number of midwives per 10,000 population - and other midwife and village characteristics. Uptake of caesarean sections, but not birth with a health professional, was linked with increased midwife density. Distance to the nearest hospital, whether the midwife was village-based or health centre-based and the length of her work experience in a community, together with women’s level of education and wealth, were also associated with birth with a health professional even when other influences were controlled for in the adjusted analysis.

A study in Matlab, Bangladesh [21] deployed trained midwives to provide antenatal and postpartum care, attend births in the home and refer complications to a maternity clinic. Midwives also worked with community health workers and TBAs. Risk of “obstetric death” [21] in the intervention area was significantly lower than in the control area in the years after the start of the programme (1987–1989) compared with the preceding years (1984–1986). The authors attribute this to improved referral for emergency obstetric care (EmOC). The top three reduced causes of death were abortion, post-partum haemorrhage and postpartum sepsis. Despite the intervention, many home births were still not attended by midwives. The authors suggest this could have been because of distance (midwives too far from the homes where the births took place), household factors (potential unwillingness of male or elder family members to make the decision to call external personnel) and/or families’ childbirth preferences.

Two studies [22], [23] reported on interventions to increase access to and use of skilled health personnel by reducing financial barriers to users. In Bangladesh, this involved a pilot of a maternal health voucher programme [23]. Eligible pregnant women were given free access to antenatal, childbirth and postnatal care with a qualified provider in a health facility or at home, and emergency care for obstetric complications. The programme also covered transportation costs and provided incentives to have births attended by a qualified provider. Providers received incentives to distribute vouchers and to cover voucher-paid services. There were significantly higher proportions of births attended by qualified providers, institutional births, at least three antenatal visits and use of post-natal care services in the programme areas compared with control areas.

In Peru, *Proyecto 2000* aimed to increase quality and use of EmOC facilities and to address financial barriers [22]. This included, from 1996, making the services culturally acceptable, and their promotion via mass media, health education and social mobilization efforts. In 1998, a maternal and child health insurance programme began, which subsidized preventive and maternity care, including childbirth in public EmOC facilities for low-income pregnant women. Acknowledging the complexity of the data and the difficulty of assessing the two programmes, the authors conclude that *Proyecto 2000* improved the quality of care in public EmOC facilities, but did not increase the probability of childbirth in these facilities. The maternal and child health insurance programme on the other hand was associated with better use of EmOC among poor women. In other words, quality improvement was not enough - financial barriers also had to be addressed.

## Discussion

Interventions to increase the use of skilled health personnel where TBAs have been providers of childbirth care have had positive effects on maternal health. Two categories of interventions emerge as likely to increase the use of skilled health personnel at birth from the evidence available at present: a) deploying skilled health personnel and b) addressing financial barriers for users.

Deploying skilled health personnel to settings where they reduce access barriers - through reduced travel or geographic distance - is associated with an increase in their presence at birth [18]–[20]. The importance of addressing financial barriers is reflected upon in two studies [22], [23]. Note, however, that these categories emerge from complex intervention conditions in which other activities were also carried out. For example, village midwives in Indonesia promoted community participation and collaboration with TBAs, but the studies do not provide information on any impact this might have had. The studies we identify in this review emphasise the need to go beyond the supply side - providing accessible skilled health personnel - and address the demand side, including cultural preferences, and the need for family and community support for maternal welfare.

Many studies did not meet our quality criteria. Intervention evaluations should be carefully designed and executed. For example, many papers claimed, without strong evidence that change had been caused by their intervention of interest. They often failed to consider other possible explanations (e.g. prevailing country-wide trends). Few studies provided any type of comparison group, which could have helped to mitigate this problem. In the absence of a comparison area, studies could also have provided high-quality qualitative evidence that the intervention was the cause of any observed changes in service use. None did so.

Some interesting case studies (12 of the 35 in the quality review) were not included in the effectiveness analysis because specific interventions could not be attributed with confidence to changes. For example, case studies described country programmes over an extended time period. However these references are included in our inventory as they can provide a deeper understanding of country commitments, strategies employed to respond to multiple factors affecting women’s use of skilled care and the complexities involved in implementing interventions to increase access to care with skilled health personnel. These complex interventions, involving multiple components across a range of domains, and often over extended time periods, are particularly challenging to evaluate because the different components of the intervention, and their outcomes, need to be independently and summatively examined [24], [25].

For the reasons stated above we are unable to comment on the effectiveness of the interventions included in the inventory. Some references in any case only describe actions taken by country programmes, rather than measure effectiveness. Interventions described in the references in the inventory include community mobilization, maternity waiting homes, addressing cultural preferences within the health services, and birth planning and complication readiness. Additional examples of addressing geographical and financial barriers, human resources development and deployment, and addressing quality can also be found, as well as efforts related to new roles for TBAs, the regulation of the midwifery profession, policy measures, and community advocacy.

From the inventory, we identify two additional themes. Firstly, many of the interventions are modified over time. This complicates evaluation, but highlights the fact that interventions need to respond to changing demands and context [26]. Secondly, sustainability of interventions is rarely assessed. Many evaluations are of pilot schemes, often conducted in a small geographical area. There is a need for research on sustainability, particularly of interventions shown to be effective in pilot studies [27], [28]. Given the variety of interventions and the low number of studies per intervention type, we do not address cost, but recognize that examining this will be crucial to inform sustainability and scale-up possibilities of effective interventions.

### Limitations

We included items indexed in either French or English, and full text in English, French, Portuguese and Spanish, so we may have excluded relevant research evidence published in other languages. We excluded studies of interventions where TBAs were trained to continue to assist at births, including training to recognise danger signs and refer for complications, but recognize that some programmes viewed TBA training as a transition strategy, particularly to increase use of EmOC services. Finally, the number of high quality studies is small, with heterogeneous outcomes. This limits our ability to conduct either a meta- or causal chain analysis. Half of the included studies in the analysis evaluate just one intervention - the village midwife programme in Indonesia - which also limits the generalizability of our conclusions.

## Supporting Information

Table S1 Inventory of included references.
(DOC)

Protocol S1 **Protocol for this systematic review.**
(DOC)
