# Supplementary material for: Increasing the Use of Skilled Health Personnel Where Traditional Birth Attendants Were Providers of Childbirth Care: A Systematic Review
Source: PLoS One. 2012 Oct 24;7(10):e47946. doi: 10.1371/journal.pone.0047946 (PMC3480459; doi:10.1371/journal.pone.0047946)
Supplement: Table S1 — Inventory of included references. (DOC) [file pone.0047946.s001.doc]

| **Country** | **Type of intervention(s)** | **Reasons for inventory only vs. inclusion in effectiveness analysis** | **Reference** |
| --- | --- | --- | --- |
| Afghanistan | - Human resources development and deployment  - Policies to increase access to services, quality of the services, community participation and midwife + TBA partnership | Inventory only: No empirical data/no analysis of relevant data | Save the Children (2008) Saving the lives of mothers and newborns in Afghanistan: Afghanistan newborn health situation analysis. Connecticut: Save the Children. 57p. [unpublished work]. |
| Australia | - Regulation of midwifery | Inventory only: No empirical data/no analysis of relevant data | Purcal N (1998) Traditional midwifery and its influence on contemporary maternity care: a brief historical review of events in New South Wales. Birth Issues 7: 58-65. |
| Bangladesh | - Human resources development | Inventory only: No empirical data/no analysis of relevant data | Ahmed T, Jakaria SM (2009) Community-based skilled birth attendants in Bangladesh: attending deliveries at home. Reprod Health Matters 17: 45-50. |
|  | - Human resources development and deployment  - Improve quality of the services | Inventory only: Covers extended time period with multiple interventions | Independent Review Team (2009) Annual Programme Review (APR), Volume I, Main consolidated report key findings, conclusions and recommendations. Bangladesh Health, Nutrition and Population Sector Programme (HNPSP). 26p. [unpublished work]. |
|  | - Human resources development | Inventory only: Does not meet quality criteria | Bhuiyan AB, Mukherjee S, Acharya S, Haider SJ, Begum F (2005) Evaluation of a Skilled Birth Attendant pilot training program in Bangladesh. Int J Gynaeco Obstet 90: 56-60. |
|  | - Human resources deployment | Inventory only: No empirical data/no analysis of relevant data | Chowdhury N (1998) Safety lessons from Matlab, Bangladesh. Plan Parent Chall 28-29. |
|  | - Human resources deployment  - Improve access to services: addressing geographical and financial barriers | Inventory only: Covers extended time period with multiple interventions | Chowdhury ME, Ahmed A, Kalim N, Koblinsky M (2009) Causes of maternal mortality decline in Matlab, Bangladesh. In: Koblinsky M, editor. [Special issue: case studies on safe motherhood](http://www.cabdirect.org:80/search.html?q=at%3A"Special+Issue%3A+Case+studies+on+safe+motherhood"). J Health Popul Nutr 27: 108-123. |
|  | - Human resources deployment  - New role for TBA: midwife + TBA partnership | Effectiveness analysis | Fauveau V, Stewart K, Khan SA, Chakraborty J (1991) Effect on mortality of community-based maternity-care programme in rural Bangladesh. Lancet 338: 1183-1186. |
|  | - Improve access to services: addressing financial barriers  - Financial and in-kind incentives to women  - Financial incentives to health workers  - Improve quality of the services | Effectiveness analysis | Hatt L, Nguyen H, Sloan N, Miner S, Magvanjav O, et al. (2010) Economic evaluation of demand-side financing (DSF) for maternal health in Bangladesh. Review, analysis and assessment of issues related to health care financing and health economics in Bangladesh. Bethesda: Abt Associates Inc. 152 p. [unpublished work]. |
|  | - Human resources development and deployment | Inventory only: No empirical data/no analysis of relevant data | Marsden P, Rahman SM, Chowdhury ME (2009) Developing options for piloting different innovative approaches to reduce the human resource gaps in maternal and neonatal health services in selected districts. DFID Health Resource Centre. 61p. [unpublished work]. |
|  | - Human resources development | Inventory only: No empirical data/no analysis of relevant data | Martz R (2008) MNH mapping: a mapping of maternal and neonatal health interventions in Bangladesh. HNP Consortium. 36p. [unpublished work]. |
|  | - Improve quality of the services  - Human resources development  - Improve access to services: addressing geographical barriers | Inventory only: No empirical data/no analysis of relevant data | Mridha MK, Anwar I, Koblinsky M (2009) Public-sector maternal health programmes and services for rural Bangladesh. In: Koblinsky M, editor. [Special Issue: Case studies on safe motherhood](http://www.cabdirect.org:80/search.html?q=at%3A"Special+Issue%3A+Case+studies+on+safe+motherhood"). J Health Popul Nutr 27: 124-138. |
|  | - Human resources development | Inventory only: No empirical data/no analysis of relevant data | Murakami I, Egami Y, Jimba M, Wakai S (2003) Training of skilled birth attendants in Bangladesh. Lancet 362: 1940. |
|  | - Human resources development and deployment  - Improve access to services: addressing financial barriers | Inventory only: Does not meet quality criteria | International Centre for Diarrhoeal Disease Research, Bangladesh (2005) Posting of trained birthing attendants: a comparison of home- and facility-based obstetric care. Health and Science Bulletin 3: 11-15. |
|  | - Human resources development and deployment | Inventory only: No empirical data/no analysis of relevant data | Tasnim S, Rahman A, Shahabuddin AK (2009) Access to skilled care at home during pregnancy and childbirth: Dhaka Bangladesh. Int Q Community Health Educ 30: 81-87. |
| Belgium | - Regulation of midwifery | Inventory only: No empirical data/no analysis of relevant data | Deroover J, Leroy F (2006) Organization of obstetrical teaching and practice in our regions (particularly in Brussels) during the XIXth century (second part). Revue Medicale de Bruxelles 27: 54-60. |
| Belize | - Regulation of midwifery | Inventory only: No empirical data/no analysis of relevant data | Blanchard DS, Bean A (2001) Healing practices of the people of Belize. Holist Nurs Pract 15: 70-78. |
| Bolivia | - Improve access to services: addressing financial barriers  - Improve quality of the services  - Community advocacy  - Cultural adaptation of institutional childbirth | Inventory only: No empirical data/no analysis of relevant data | Heichelheim J, Koblinsky M (1997) Learning and action in the first decade - the MotherCare experience. MotherCare Matters 6: 1-31. |
|  | - New role for TBA: paid to refer pregnant women to health services  - Cultural adaptation of institutional childbirth | Inventory only: No empirical data/no analysis of relevant data | Onuki D (2002) Humanization of childbirth in Bolivia. Midwifery Today Int Midwife: 54-55. |
|  | - Human resources development  - Financial incentives to women  - Cultural adaptation of institutional childbirth | Inventory only: No empirical data/no analysis of relevant data | Pelling J (2009) Bolivia: Training midwives to treat indigenous mothers with respect. United Nations Population Fund. [unpublished work]. Accessed 30 April 2012: <http://www.unfpa.org/public/News/pid/2614> . |
| Burkina Faso | - New role for TBA: health services + TBA partnership | Inventory only: No empirical data/no analysis of relevant data | Ministère de la Santé, Secrétariat Général, Direction Générale de la Santé, Direction de la Santé de la Famille (2007) Guide d’orientation pour la mise en œuvre du nouveau rôle des accoucheuses villageoises.  Ministère de la Santé, Secrétariat Général, Direction Générale de la Santé, Direction de la Santé de la Famille. 17p. [unpublished work]. |
|  | - Community advocacy | Inventory only: No empirical data/no analysis of relevant data | Ministère de la Santé, Secrétariat Général, Direction Générale de la Santé, Direction de la Santé de la Famille (2007) Guide pour la mise en place et le fonctionnement des cellules de gestion des urgencies obstetriciales et neonatales au niveau village. Ministère de la Santé, Secrétariat Général, Direction Générale de la Santé, Direction de la Santé de la Famille. 9p. [unpublished work]. |
| Cambodia | - Human resources development  - Financial incentives for institutional childbirth  - Improve access to services: addressing financial barriers  - New role for TBA: paid to refer pregnant women to health services | Inventory only: No empirical data/no analysis of relevant data | Chatterjee P (2005) Cambodia tackles high maternal mortality. Lancet 366: 281-282. |
|  | - Improve access to services: addressing financial barriers  - Financial incentives to health facility and health workers | Inventory only: Does not meet quality criteria | Ir P, Horeman D, Narin S, Van Damme W (2008) Improving access to safe delivery for poor pregnant women: a case study of vouchers plus health equity funds in three health districts in Cambodia. In: Richard F, Witter S, De Brouwere V, editors. Reducing financial barriers to obstetric care in low-income countries. Antwerp: ITGPress. pp 225-255. |
|  | - Improve access to services: addressing financial barriers  - Financial incentives to health facility and health workers | Inventory only: Does not meet quality criteria | Ir P, Horemans D, Souk N, Van Damme W (2010) Using targeted vouchers and health equity funds to improve access to skilled birth attendants for poor women: a case study in three rural health districts in Cambodia. BMC Pregnancy and Childbirth 10: 1-11. |
|  | - Community advocacy | Inventory only: Does not meet quality criteria | Skinner J, Rathavy T (2009) Design and evaluation of a community participatory, birth preparedness project in Cambodia. Midwifery 25: 738-743. |
| Canada | - Regulation of midwifery | Inventory only: No empirical data/no analysis of relevant data | Fynes MT (1994) The legitimation of midwifery in Ontario, 1960-1987: Master thesis..University of Toronto. 180p. [unpublished work]. |
| Chile | - Human resources development  - Improve quality of the services  - Improve access to services: addressing financial barriers | Inventory only: No empirical data/no analysis of relevant data | Campos MS (2008) Clinical and medical assistance in childbirth: trajectory of national and international influences, Santiago, Chile, 1900-1950. Can Bull Med Hist 25: 193-224. |
| China | - Human resources development  - Regulation of midwifery | Inventory only: No empirical data/no analysis of relevant data | Harris A, Belton S, Barclay L, Fenwick J (2009) Midwives in China: 'jie sheng po' to 'zhu chan shi'. Midwifery 25: 203-212. |
|  | - Improve access to services: addressing geographical and financial barriers  - TBAs prohibited  - New role for TBA: advocacy | Inventory only: No empirical data/no analysis of relevant data | Harvey TA (2003) The jie sheng po of China. Midwifery Today 66: 53-54. |
|  | - Improve access to services: addressing geographical barriers | Inventory only: No empirical data/no analysis of relevant data | Shanghai Municipal Health Bureau (1985) Maternal and child health (MCH). Control of neonatal tetanus. Weekly Epidemiological Record 60: 5-6. |
|  | - Improve access to services: addressing financial barriers  - Human resources development  - Improve quality of the services | Inventory only: Covers extended time period with multiple interventions | Institute for Health Science (2003) Yunnan, China 1980-1999. In: Koblinsky MA, editor. Reducing maternal mortality: learning from Bolivia, China, Egypt, Honduras, Indonesia, Jamaica and Zimbabwe. Washington DC: Human Development Network, Health, Nutrition and Population Series, World Bank. pp 41-50. |
| Costa Rica | - Improve access to services: addressing geographical and financial barriers  - Human resources deployment  - TBAs prohibited | Inventory only: No empirical data/no analysis of relevant data | Jenkins GL (2003) Burning bridges: policy, practice, and the destruction of midwifery in rural Costa Rica. Soc Sci Med 56: 1893-1909. |
| Ecuador | - Cultural adaptation of institutional childbirth  - Improve access to services: addressing financial barriers | Inventory only: No empirical data/no analysis of relevant data | Vivar SC (2007) Ecuador addresses cultural issues for pregnant women. Lancet 370: 1302. |
| England | - Improve access to services: addressing geographical and financial barriers  - Improve quality of the services  - Human resources development  - Regulation of midwifery | Inventory only: Covers extended time period with multiple interventions | Beier LM (2004) Expertise and control: Childbearing in three twentieth-century working-class Lancashire communities. Bull Hist Med 78: 379-409. |
| Eritrea | - Improve access to services: addressing geographical barriers  - Human resources development  - New role for TBA: accompanying women to health services | Inventory only: Does not meet quality criteria | Andemichael G, Haile B, Kosia A, Mufunda J. Maternity waiting homes: A panacea for maternal/neonatal conundrums in Eritrea. Journal of Eritrean Medical Association 18-21. |
| Ghana | - Improve access to services: addressing financial barriers | Inventory only: No empirical data/no analysis of relevant data | Asante FA, Chikwama C, Daniels A, Armar-Klemesu M (2007) Evaluating the economic outcomes of the policy of fee exemption for maternal delivery care in Ghana. Ghana Med J 41: 110-117. |
|  | - Improve access to services: addressing financial barriers | Inventory only: Does not meet quality criteria | Penfold S, Harrison E, Bell J, Fitzmaurice A (2007) Evaluation of the delivery fee exemption policy in Ghana: population estimates of changes in delivery service utilization in two regions. Ghana Med J 41: 100-109. |
|  | - Improve access to services: addressing financial barriers | Inventory only: No empirical data/no analysis of relevant data | Witter S, Kusi A, Aikins M (2007) Working practices and incomes of health workers: evidence from an evaluation of a delivery fee exemption scheme in Ghana. Hum Resour Health 5: 2. |
|  | - Improve access to services: addressing financial barriers | Inventory only: No empirical data/no analysis of relevant data | Witter S, Adjei S, Armar-Klemesu M, Graham W (2009) Providing free maternal health care: ten lessons from an evaluation of the national delivery exemption policy in Ghana. Glob Health Action DOI 10.3402. |
| Guatemala | - Human resources development  - Improve access to services: addressing geographical barriers | Inventory only: No empirical data/no analysis of relevant data | Houston J (1998) Midwife. Guatemala: one step at a time. Midwifery Today 47: 56, 57, 59. |
|  | - New role for TBA: providing support at institutional birth | Inventory only: No empirical data/no analysis of relevant data | JHPIEGO (2002) The traditional birth attendant: linking communities and services. Baltimore: JHPIEGO. 2p. [unpublished work]. |
| Haiti | - Improve access to services: addressing geographical barriers | Inventory only: Does not meet quality criteria | Shaffer S, Fryzelka D, Obenhaus C, Wickstrom E (2007) Improving maternal healthcare access and neonatal survival through a birthing home model in rural Haiti. Soc Med 2: 177-185. |
| Honduras | - Improve access to services: addressing geographical barriers | Inventory only: No empirical data/no analysis of relevant data | República de Honduras, Secretaría de Salud (2009) Hogares maternos: lineamientos para su implementación y desarrollo en Honduras. República de Honduras. Secretaría de Salud. 15p. [unpublished work]. |
|  | - Improve access to services: addressing geographical barriers  - Improve quality of services  - Financial incentives to women  - Community advocacy  - New role for TBA: accompanying women to health services | Inventory only: No empirical data/no analysis of relevant data | Secretaria de Salud de Honduras, Sub Secretaria de Riesgos Poblacionales, Sub Secretaria de Redes de Servicios. RAMNI: reducción acelerada de la mortalidad maternal y de la niñez 2008-2015. Secretaria de Salud de Honduras, Sub Secretaria de Riesgos Poblacionales, Sub Secretaria de Redes de Servicios. 55p [unpublished work]. |
|  | - Human resources deployment  - Improve quality of services  - Improve access to services: addressing geographical barriers | Inventory only: No empirical data/no analysis of relevant data | Rashid S (2004) Success in Honduras. Countdown 2015: sexual and reproductive health and rights for all: 82. |
| India | - Improve access to services: addressing financial barriers | Inventory only: Does not meet quality criteria | Bhat R, Mavalankar DV, Singh PV, Singh N (2009) Maternal healthcare financing: Gujarat’s Chiranjeevi scheme and its beneficiaries. In: Koblinsky M, editor. [Special issue: Case studies on safe motherhood](http://www.cabdirect.org:80/search.html?q=at%3A"Special+Issue%3A+Case+studies+on+safe+motherhood"). J Health Popul Nutr 27: 249-258 |
|  | - Improve quality of the services  - Financial incentives to health workers, TBAs and women  - New role for TBA: health services + TBA partnership | Inventory only: No empirical data/no analysis of relevant data | Ministry of Health & Family Welfare. Government of India. Janani Suraksha Yojana: guidelines for implementation. Ministry of Health & Family Welfare. Government of India. 26p. [unpublished work]. |
|  | - Improve access to services: addressing financial barriers  - Financial incentives to obstetricians  - Human resources development and deployment  - Improve quality of the services | Inventory only: Covers extended time period with multiple interventions | Mavalankar DV, Vora KS, Ramani KV, Raman P, Sharma B, et al. (2009) Maternal health in Gujarat, India: a case study. In: Koblinsky M, editor. [Special Issue: Case studies on safe motherhood](http://www.cabdirect.org:80/search.html?q=at%3A"Special+Issue%3A+Case+studies+on+safe+motherhood"). J Health Popul Nutr 27: 235-248. |
|  | - Improve quality of services | Inventory only: No empirical data/no analysis of relevant data | Mutharayappa R (2005) A study of maternal health services in three districts of Karnataka. J Indian Anthropol Soc 40: 87-99 |
|  | - Human resources development and deployment  - Improve quality of the services  - Financial incentives to health workers and women  - Improve social support at institutional childbirth  - Improve access to services: addressing geographical barriers | Inventory only: No empirical data/no analysis of relevant data | Padmanaban P, Raman PS, Mavalankar DV (2009) Innovations and challenges in reducing maternal mortality in Tamil Nadu, India. In: Koblinsky M, editor. [Special issue: Case studies on safe motherhood](http://www.cabdirect.org:80/search.html?q=at%3A"Special+Issue%3A+Case+studies+on+safe+motherhood"). J Health Popul Nutr 27: 202-219. |
|  | - Improve access to services: addressing geographical and financial barriers  - Human resources development  - Improve the quality of services  - Community advocacy | Inventory only: Covers extended time period with multiple interventions | Prakasamma M (2009) Maternal mortality-reduction programme in Andhra Pradesh. In: Koblinsky M, editor. [Special issue: Case studies on safe motherhood](http://www.cabdirect.org:80/search.html?q=at%3A"Special+Issue%3A+Case+studies+on+safe+motherhood"). J Health Popul Nutr 27: 220-234. |
|  | - Human resources development and deployment  - Improve the quality of services  - Improve access to services: addressing geographical barriers  - Financial incentives to midwives and women | Inventory only: Covers extended time period with multiple interventions | Vora KS, Mavalankar DV, Ramani KV, Upadhyaya M, Sharma B et al. (2009) Maternal health situation in India: a case study. In: Koblinsky M, editor. [Special issue: Case studies on safe motherhood](http://www.cabdirect.org:80/search.html?q=at%3A"Special+Issue%3A+Case+studies+on+safe+motherhood"). J Health Popul Nutr 27: 184-201. |
| Indonesia | - Human resources development and deployment  - Improve access to services: addressing geographical and financial barriers  - New role for TBA: midwife + TBA partnership | Inventory only: No empirical data/no analysis of relevant data | World Bank (2010) “…and then she died”: Indonesia maternal health assessment. World Bank. 80p. [unpublished work]. |
|  | - Human resources development and deployment | Effectiveness analysis | Achadi E, Scott S, Pambudi ES, Makowiecka K, Marshall T, et al. (2007) Midwifery provision and uptake of maternity care in Indonesia. Trop Med Int Health 12: 1490–1497. |
|  | - Community advocacy  - Improve access to services: addressing geographical and financial barriers  - Improve quality of the services | Inventory only: Does not meet quality criteria | Fachry A, Sofiarini R Developing the Desa Siaga program in NTB province: evaluation of the Desa Siap Antar Jaga (DSAJ) program in villages supported by GTZ SISKES. 26p. [unpublished work]. |
|  | - Human resources development and deployment | Effectiveness analysis | Frankenberg E, Buttenheim A, Sikoki B, Suriastini W (2009) Do women increase their use of reproductive health care when it becomes more available? Evidence from Indonesia. Stud Fam Plann 40: 27-38. |
|  | - Human resources development and deployment  - Improve access to services: addressing geographical barriers | Inventory only: No empirical data/no analysis of relevant data | Geefhuysen CJ (1999) Safe Motherhood in Indonesia: a task for the next century. In Berer M, Sundari Ravindran TK, editors. Safe motherhood initiatives: critical issues. Oxford: Blackwell Science. pp 62-72. |
|  | - Human resources deployment  - Improve access to services: addressing geographical barriers  - TBAs prohibited for childbirth | Inventory only: Does not meet quality criteria | Hallberg TL (1999) Rural Javanese midwives: accommodating and resisting biomedicine: PhD thesis. University of Oregon. 391p. [unpublished work]. |
|  | - New role for TBA: midwife + TBA partnership | Inventory only: No empirical data/no analysis of relevant data | AusAID (2003) Healthy mothers, healthy babies. Child survival project. Milestone 53 evaluation report. Draft. AusAID. 3p [unpublished work]. |
|  | - Improve access to services: addressing financial barriers  - Community advocacy  - New role for TBA: midwife + TBA partnership | Inventory only: No empirical data/no analysis of relevant data | Henderson C (2009) Sipakatau: A holistic approach to maternal healthcare. Just Change 14: 21. |
|  | - Improve access to services: addressing financial barriers  - Human resources development and deployment | Inventory only: Covers extended time period with multiple interventions | Koblinsky MA (2003) Indonesia 1990-1999. In: Koblinsky MA, editor. Reducing maternal mortality: learning from Bolivia, China, Egypt, Honduras, Indonesia, Jamaica and Zimbabwe. Washington DC: Human Development Network, Health, Nutrition and Population Series, World Bank. pp 113-121. |
|  | - Human resources development and deployment  - Improve quality of the services  - Community advocacy | Inventory only: No empirical data/no analysis of relevant data | Kwast BE, Koblinsky MA (1995) Starting maternity care programmes in developing countries to reduce maternal mortality. Contemp Rev Obstet Gynaecol 7: 220-225. |
|  | - Improve quality of the services  - New role for TBA: midwife + TBA partnership  - Human resources development | Inventory only: No empirical data/no analysis of relevant data | UNICEF Indonesia (2007) Local Area Monitoring and Tracking (LAMAT). UNICEF Indonesia. 3p. [unpublished work]. |
|  | - Human resources development  - New role for TBA: midwife + TBA partnership | Inventory only: No empirical data/no analysis of relevant data | Niehof A (2010) The changing role of the traditional birth attendant in Indonesia. 15p. [unpublished work]. |
|  | - Improve access to services: addressing geographical barriers  - Human resources deployment | Inventory only: No empirical data/no analysis of relevant data | Parker E, Roestam A (2003) The Bidan di Desa program: a literature and policy review. Maternal and Neonatal Health Program (MNH), JHPIEGO Corporation. 42p. [unpublished work]. |
|  | - Human resources development and deployment  - New role for TBA: midwife + TBA partnership  - Community advocacy  - Improve access to services: addressing financial barriers | Inventory only: Covers extended time period with multiple interventions | Ronsmans C, Endang A, Gunawan S, Zazr A, McDermott J, et al. (2001) Evaluation of a comprehensive home-based midwifery programme in South Kalimantan, Indonesia. Trop Med Int Health 6: 799-810. |
|  | - Human resources development and deployment | Effectiveness analysis | Shrestha R (2007) Family planning, community health interventions and the mortality risk of children in Indonesia: PhD thesis. The Ohio State University. 108 p. [unpublished work]. |
|  | - Human resources deployment  - New role for TBA: midwife + TBA partnership | Inventory only: No empirical data/no analysis of relevant data | Stein EA (2007) Midwives, Islamic morality and village biopower in Post-Suharto Indonesia. Body & Society 13: 55-77. |
|  | - Improve access to services: addressing financial barriers  - Human resources development and deployment | Inventory only: Covers extended time period with multiple interventions | Tan ESM (2006) Case study 2: vouchers for midwife services in Pemalang District, Central Java. In: World Bank, editor. Making services work for the Poor: nine case studies from Indonesia. 41-58. |
|  | - Community advocacy | Inventory only: No empirical data/no analysis of relevant data | Deutsche Gesellschaft für Technische Zusammenarbeit (GTZ) GmbH (2009) Toolkit: community empowerment in MNH. Eschborn: Deutsche Gesellschaft für Technische Zusammenarbeit (GTZ) GmbH. 197p. [unpublished work]. |
|  | - New role for TBA: midwife + TBA partnership | Inventory only: No empirical data/no analysis of relevant data | UNICEF Indonesia (2007) Traditional Birth Attendant – Midwife Partnership. UNICEF Indonesia. 3p. [unpublished work]. |
|  | - New role for TBA: midwife + TBA partnership  - Community advocacy | Inventory only: No empirical data/no analysis of relevant data | Weber M (2010) Letter from a best friend (Surat dari Sahabat). 2p. [personal communication]. |
| Jamaica | - Regulation of midwifery  - Human resources development and deployment  - Improve access to services: addressing geographical barriers  - Improve quality of the services | Inventory only: No empirical data/no analysis of relevant data | McCaw-Binns A (2005) Safe Motherhood in Jamaica: from slavery to self-determination. Paediatr Perinat Epidemiol 19: 254-261. |
|  | - Regulation of midwifery  - Human resources development | Inventory only: No empirical data/no analysis of relevant data | Sargent C, Rawlins J (1992) Transformations in maternity services in Jamaica. Soc Sci Med 35: 1225-1232. |
| Kenya | - Improve quality of the services  - Community advocacy  - Improve access to services: addressing geographical barriers | Inventory only: No empirical data/no analysis of relevant data | Olson J. 10p. [personal communication]. |
| Lao People's Democratic Republic | - Improve access to services: addressing geographical barriers | Inventory only: No empirical data/no analysis of relevant data | Chithtalath SA, Earth B (2001) From the forest to the clinic: changing birth practice among the Katang, Lao. Reprod Health Matters 9: 99-104. |
| Malaysia | - TBAs prohibited | Inventory only: No empirical data/no analysis of relevant data | Utusan Konsumer (2002) Bidan - traditional Malay midwives fighting for survival. Midwifery Matters 92: 16. |
|  | - Regulation of midwifery  - Human resources development  - Improve access to services: addressing geographical barriers | Inventory only: No empirical data/no analysis of relevant data | Chen PC (1973) The medical auxiliary in rural Malaysia. Lancet 1: 983-985. |
|  | -Human resources deployment  - New role for TBA: midwife + TBA partnership | Inventory only: No empirical data/no analysis of relevant data | Chen PCY (1977) Incorporating the traditional birth attendant into the health team: the Malaysian example. Trop Geogr Med 29: 192-196. |
|  | - Human resources development and deployment  - Improve access to services: addressing geographical barriers | Inventory only: No empirical data/no analysis of relevant data | Chen PCY (1977) Providing maternal and child care in rural Malaysia. Trop Geogr Med 29: 441-448. |
| Mexico | - Financial incentives for institutional childbirth  - National advocacy | Inventory only: No empirical data/no analysis of relevant data | Alzugaray M (2004) The birth of a collaboration. Midwifery Today 69: 48-50. |
|  | - New role for TBA: Doctor + TBA partnership  - Cultural adaptation of institutional childbirth  - Human resources development | Inventory only: No empirical data/no analysis of relevant data | Braine T (2008) Mexico's midwives enter the mainstream. Bull World Health Organ 86: 244-245. |
|  | - New role for TBA: midwife + TBA partnership  - Cultural adaptation of institutional childbirth | Inventory only: No empirical data/no analysis of relevant data | Cao Romero L (1997) Bridges of understanding: midwifery in Mexico. Midwifery Today 43: 52-53. |
|  | - Improve access to services: addressing geographical barriers  - Regulation of midwifery  - Human resources development  - New role for TBA: midwife + TBA partnership | Inventory only: No empirical data/no analysis of relevant data | Davis-Floyd R (2001) La partera profesional: articulating identity and cultural space for a new kind of midwife in Mexico. Med Anthropol 20: 185-243. |
|  | - New role for TBA: providing support at institutional birth | Inventory only: Does not meet quality criteria | Smid M, Hernandez D, Campero L, Cragin L, DeMaria L, et al. (2008) Partera-Doula: Exploring the integration of traditional midwives into the Mexican public hospital system to provide labor and delivery support. American Public Health Association 136^th^ Annual Meeting and Expo. California. 6p. [unpublished work]. |
| Mozambique | - Human resources development  - Improve access to services: addressing geographical barriers | Inventory only: No empirical data/no analysis of relevant data | Raisler J (1984) Nurse-midwifery in a developing country: maternal and child health in Mozambique. J Nurse Midwifery 29: 399-402. |
| Multi-country | - Human resources development  - Improve quality of the services  - Community advocacy | Inventory only: No empirical data/no analysis of relevant data | Family Care International (2005) FCI initiative boosts skilled birthing care. Lives - The newsletter of the partnership for safe motherhood and newborn health 1:11. |
|  | - Human resources development  - Regulation of midwifery | Inventory only: No empirical data/no analysis of relevant data | De Brouwere V, Tonglet R, van Lerberghe W (1998) Strategies for reducing maternal mortality in developing countries: what can we learn from the history of the industrialized West? Trop Med Int Health 3: 771-782. |
|  | - Regulation of midwifery | Inventory only: No empirical data/no analysis of relevant data | De Brouwere V (2007) The comparative study of maternal mortality over time: the role of the professionalisation of childbirth. Soc Hist Med 20: 541-562. |
|  | - Human resources development  - TBAs prohibited  - Implementation of sanctions  - Improve access to services: addressing geographical barriers | Inventory only: No empirical data/no analysis of relevant data | Jett J (1977) The role of traditional midwives in modern health practices in West Africa and Central Africa. Washington, DC: US Agency for International Development. [unpublished work]. |
|  | - Human resources development and deployment  - Improve access to services: addressing geographical and financial barriers  - Improve quality of the services  - New role for TBA: support at childbirth with health workers | Inventory only: No empirical data/no analysis of relevant data | Koblinsky MA, Campbell O, Heichelheim J (1999) Organizing delivery care: what works for safe motherhood? Bull World Health Organ 77: 399-406. |
|  | - Human resources development  - Improve access to services: addressing geographical and financial barriers | Inventory only: Covers extended time period with multiple interventions | Liljestrand J, Pathmanathan I (2004) Reducing maternal mortality: can we derive policy guidance from developing country experiences? J Public Health Policy 25: 299-314. |
|  | - TBAs prohibited | Inventory only: No empirical data/no analysis of relevant data | Owen M (1983) The traditional birth attendant and the law. World Health Forum 4: 291-298. |
|  | - Improve access to services: addressing geographical and financial barriers  - Community advocacy  - Improve quality of the services  - Regulation of midwifery  - Intersectoral approaches  - Human resources development and deployment  - New role for TBA: midwife + TBA partnership | Inventory only: Covers extended time period with multiple interventions | Pathmanathan I, Liljestrand J, Martins JM, Rajapaksa LC, Lissner C, et al. (2003) Investing in maternal health: learning from Malaysia and Sri Lanka. Human Development Network, Health, Nutrition and Population Series, World Bank 2003. |
|  | - Human resources development | Inventory only: No empirical data/no analysis of relevant data | van Lerberghe W, De Brouwere V (2001) Of blind alleys and things that have worked: history’s lessons on reducing maternal mortality. In De Brouwere V, van Lerberghe W, editors. Safe Motherhood strategies: a review of the evidence. Antwerp: ITGPress. pp 7-33. |
| Myanmar | - Community advocacy  - Human resources development  - New role for TBA: health services + TBA partnership  - Improve access to services: addressing geographical barriers | Inventory only: No empirical data/no analysis of relevant data | Myanmar Ministry of Health (2010). 15p. [personal communication]. |
|  | - Human resources development and deployment | Inventory only: No empirical data/no analysis of relevant data | Parker T (1987) Nursing Aid. Midwives of independent means. Nurs Times 83: 42-43. |
| Nepal | - Human resources development  - Improve access to services: addressing geographical barriers | Inventory only: No empirical data/no analysis of relevant data | Frazer PE (1995) Midwifery in Nepal. British Journal of Midwifery 3: 469-472. |
| New Zealand | - Regulation of midwifery | Inventory only: No empirical data/no analysis of relevant data | Stojanovic J (2008) Midwifery in New Zealand 1904-1971. Contemporary Nurse 30: 156-167. |
| Nigeria | - New role for TBA: health services + TBA partnership | Inventory only: No empirical data/no analysis of relevant data | Federal Government of Nigeria, Federal Ministry of Health (2010) Changing roles – refocusing traditional birth attendant towards accelerated reduction of maternal and newborn mortality in Nigeria, Concept Paper. Federal Government of Nigeria, Federal Ministry of Health. 14p. [unpublished work]. |
|  | - Human resources development  - Improve access to services: addressing geographical barriers  - Financial incentives to midwives | Inventory only: No empirical data/no analysis of relevant data | Harrison KA (2003) Reproductive health struggles in Nigeria. Lancet 362: 582. |
|  | - Improve access to services: addressing geographical barriers | Inventory only: Does not meet quality criteria | Isenalumbe AE (1990) Integration of traditional birth attendants into primary health care. World Health Forum 11: 192-198. |
|  | - Improve access to services: addressing geographical and financial barriers | Inventory only: Does not meet quality criteria | Nwakoby BN (1992) The influence of new maternal care facilities in rural Nigeria. Health Policy Plan 7: 269-278. |
| Palestine | - Improve access to services: addressing financial barriers | Inventory only: No empirical data/no analysis of relevant data | Wick L (2002) Birth at the checkpoint, the home or the hospital? Adapting to the changing reality in Palestine. In: International Confederation of Midwives 2002. Midwives and women working together for the family of the world: ICM-ROM. 11p. [unpublished work]. |
| Peru | - Cultural adaptation of institutional childbirth | Inventory only: Does not meet quality criteria | Gabrysch S, Lema C, Bedrinana E, Bautista MA, Malca R, et al. (2009) Cultural adaptation of birthing services in rural Ayacucho, Peru. Bull World Health Organ 87: 724-729. |
|  | - Cultural adaptation of institutional childbirth  - Implementation of sanctions | Inventory only: No empirical data/no analysis of relevant data | Ingar C (2008) Midwifery & birthing: women in Peru. Midwifery Today 85: 51. |
|  | - Human resources deployment  - Regulation of midwifery  - Implementation of sanctions  - Cultural adaptation of childbirth  - New role for TBA: doctor + TBA partnership  - Improve access to services: addressing geographical barriers | Inventory only: No empirical data/no analysis of relevant data | Madison R (2002) Peru: midwifery on high. Midwifery Today 61: 53-54. |
|  | - Improve access to services: addressing financial barriers  - Improve quality of the services  - Cultural adaptation of institutional childbirth | Effectiveness analysis | McQuestion MJ, Velasquez A (2006) Evaluating program effects on institutional delivery in Peru. Health Policy 77: 221-232. |
| Senegal | - Policy to improve access to services: addressing financial barriers | Inventory only: Does not meet quality criteria | Witter S, Dieng T, Mbengue D, Moreira I, De Brouwere V (2010) The national free delivery and caesarean policy in Senegal: evaluating process and outcomes. Health Policy Plan 25: 384-392. |
| Southern Sudan | - New role for TBA: midwife + TBA partnership  - Financial incentives to TBAs | Inventory only: No empirical data/no analysis of relevant data | Government of Southern Sudan, Ministry of Health. Maternal, Neonatal and Reproductive Health strategy 2009-2012. draft. Government of Southern Sudan, Ministry of Health. 50p. [unpublished work]. |
| Sri Lanka | - Human resources development | Inventory only: No empirical data/no analysis of relevant data | West KM (1981) Sri Lanka: exploring the use of the TBA as a low-cost means for family health. In: Mangay-Maglacas A, Pizurki H, editors. The traditional birth attendant in seven countries, case studies in utilization and training. Geneva: WHO. pp 97-130. |
| Sudan | - Regulation of midwifery | Inventory only: No empirical data/no analysis of relevant data | Bell H (1998) Midwifery training and female circumcision in the inter-war Anglo-Egyptian Sudan. J Afr Hist39: 293-312. |
|  | - Human resources development  - Regulation of midwifery | Inventory only: No empirical data/no analysis of relevant data | El Hakim S (1981) Sudan: replacing TBAs by village midwives. In: Mangay-Maglacas A, Pizurki H, editors. The traditional birth attendant in seven countries, case studies in utilization and training. Geneva: WHO. pp 131-166. |
| Thailand | - Human resources development | Inventory only: No empirical data/no analysis of relevant data | Maclean GD (2003) The challenge of preparing and enabling 'skilled attendants' to promote safer childbirth. Midwifery 19:163-169. |
| Togo | - Human resources development and deployment | Inventory only: No empirical data/no analysis of relevant data | Naassou ML (1974) Rural midwives in Togo: Presented at the ICM/USAID Project Francophone West African Working Party.15 p. [unpublished work]. |
| Uganda | - Human resources development for links between the community and health services | Inventory only: No empirical data/no analysis of relevant data | Edwidge K (2009) Traditional midwives and maternal morbidity and mortality in countries with low resources. Midwifery Today 89: 55-56. |
|  | - Birth plans | Inventory only: Does not meet quality criteria | Mulogo EM, Witte K, Bajunirwe F, Nabukera SK, Muchunguzi C, et al. (2006) Birth plans and health facility-based delivery in rural Uganda. East Afr Med J 83: 74-83. |
|  | - TBAs prohibited for childbirth  - New role for TBA: advocacy and accompanying women to health services | Inventory only: No empirical data/no analysis of relevant data | Murigi SF (2010) Should Uganda ban traditional birth attendants? Guardian. Accessed 12 April 2012: <http://www.guardian.co.uk/katine/katine-chronicles-blog/2010/mar/30/traditional-birth-attendants-ban> |
| United States of America | - Regulation of midwifery | Inventory only: No empirical data/no analysis of relevant data | Berg J (1999) Midwives as the quintessential barefoot doctors. Midwifery Today 52: 18-23. |
|  | - Regulation of nurse-midwifery | Inventory only: No empirical data/no analysis of relevant data | Ettinger LE (1999) The birth of a new professional: The nurse-midwife in the United States, 1925-1955: PhD thesis. Rochester: University of Rochester. 325p. [unpublished work]. |
|  | - Regulation of midwifery | Inventory only: No empirical data/no analysis of relevant data | Fraser GJ. Afro-American midwives, biomedicine and the state: an ethnohistorical account of birth and its transformation in rural Virginia: PhD thesis. Baltimore: Johns Hopkins University. 492p. [unpublished work]. |
|  | - Regulation of midwifery | Inventory only: No empirical data/no analysis of relevant data | Halperin J (1994) Midwifery in modern age: issue in Illinois centers on whether to license lay midwives. Illinois Issues 20: 24-26. |
|  | - Regulation of midwifery | Inventory only: No empirical data/no analysis of relevant data | Jones ZO (2004) Knowledge systems in conflict: the regulation of African American midwifery. Nurs Hist Rev12: 167-184. |
|  | - Regulation of midwifery | Inventory only: No empirical data/no analysis of relevant data | Lawn-Day GA (1994) Using institutionalized social movements to explain policy implementation failure: The case of midwifery: PhD thesis. Norman: University of Oklahoma. 399p. [unpublished work]. |
|  | - Regulation of midwifery | Inventory only: No empirical data/no analysis of relevant data | Lay MM (2003) Midwifery on trial: balancing privacy rights and health concerns after Roe v. Wade. Q J Speech 89: 60-77. |
|  | - Regulation of midwifery | Inventory only: No empirical data/no analysis of relevant data | McIntosh KE (1989) Regulation of midwives as home birth attendants. Boston Coll Law Rev30: 477-522. |
|  | - Regulation of midwifery | Inventory only: No empirical data/no analysis of relevant data | Morrison SM, Fee E (2010) Nothing to work with but cleanliness: the training of African American traditional midwives in the South. Am J Public Health 100: 238-239. |
|  | - Regulation of midwifery | Inventory only: No empirical data/no analysis of relevant data | Ortiz FM (2005) History of midwifery in New Mexico: partnership between curandera-parteras and the New Mexico Department of Health. Journal Midwifery Women's Health 50: 411-7. |
|  | - Regulation of midwifery | Inventory only: No empirical data/no analysis of relevant data | Tilghman J (1992) A study of African American lay midwifery experiences in rural South Carolina, 1950-70: PhD thesis. Coral Gables: University of Miami. 152p. [unpublished work]. |
|  | - Regulation of midwifery | Inventory only: No empirical data/no analysis of relevant data | Walsh LV (1992) "A special vocation": Philadelphia midwives, 1910-1940: PhD thesis. University of Pennsylvania. 240p. [unpublished work]. |
|  | - Regulation of midwifery | Inventory only: No empirical data/no analysis of relevant data | Weitz R, Sullivan D (1985) Licensed lay midwifery and the medical model of childbirth. Sociol Health Ill 7: 36-54. |
