# Supplementary material for: Increasing the Use of Skilled Health Personnel Where Traditional Birth Attendants Were Providers of Childbirth Care: A Systematic Review
Source: PLoS One. 2012 Oct 24;7(10):e47946. doi: 10.1371/journal.pone.0047946 (PMC3480459; doi:10.1371/journal.pone.0047946)
Supplement: Protocol S1 — Protocol for this systematic review. (DOC) [file pone.0047946.s002.doc]

**Research questions**

a) What interventions have been undertaken to increase the use of skilled health personnel in settings where traditional birth attendants were providers of childbirth care?

b) What were the effects of these interventions?

**Background**

Every day 1000 women die from pregnancy or childbirth related complications . In 2010, 287,000 maternal deaths worldwide were estimated to have occurred, most of them preventable . The major causes of death of pregnant women are severe bleeding, infections, hypertensive disorders in pregnancy and obstructed labour . Better access to skilled health personnel for childbirth is a priority strategy and a key indicator for Millennium Development Goal (MDG) 5a to improve maternal health.

In the 1970s, international organizations, including the World Health Organization (WHO), invested in the training of traditional birth attendants (TBAs) for childbirth care. However, in 1992, WHO, the United Nations Fund for Population and the United Nations Children's Fund issued a joint statement promoting the training of TBAs as a way of increasing access to childbirth care in view of the shortage of professional midwives and institutional facilities . Subsequently evidence has shown that training TBAs for childbirth care had not reduced maternal mortality . By 1997, international support for the continued training of TBAs was withdrawn, and attention turned towards “skilled birth attendant” and skilled care [[1]](#footnote-2) during pregnancy, childbirth and the immediate postpartum/postnatal period . It was considered that the continued investments in strategies based solely on training TBAs was diverting funds from the more important strategy of achieving skilled care for every birth. Nonetheless TBAs continued to be viewed as an important and valued resource that could and should play an important role in supporting improvements to maternal and newborn health . In those contexts where access to services was limited, TBA training could still be considered to be an important strategy for the transition to skilled care .

In 2004 a joint statement was issued by WHO, the International Confederation of Midwives and the International Federation of Gynaecology and Obstetricians defining the critical role of the skilled attendant . A skilled attendant was defined as “an accredited health professional - such as a midwife, doctor or nurse - who has been educated and trained to proficiency in the skills needed to manage normal (uncomplicated) pregnancies, childbirth and the immediate postnatal period, and in the identification, management and referral of complications in women and newborns.” . The statement identified the skills and abilities required for a skilled attendant, referred to in this protocol as skilled health personnel, and encouraged countries to work with TBAs to define new roles within the skilled attendant strategy and to ensure good working relations between TBAs, skilled attendants, and staff in referral facilities.

As the deadline of 2015 to meet MDG5 approaches, it is increasingly important to understand what interventions are effective in increasing births with skilled health personnel. The current systematic review intends to identify and better understand the interventions implemented in different countries to increase the use of skilled health personnel in settings where TBAs were providers of childbirth care, and to summarize the outcomes of the different interventions.

**Objectives**

The objectives of this review are to describe:

- interventions to move away from TBAs as providers of childbirth care; and

- what has worked to increase use of skilled health personnel where TBAs were providers of childbirth care.

**Methods**

Criteria for inclusion of references in this review

*Types of studies*

Due to the great diversity in conditions and settings of public health interventions, some authors consider that review questions cannot specify the type of study design to be included. We will not exclude studies based on study design alone. We will include published and unpublished studies.

*Types of participants*

This review examines two main types of participant: TBAs: “a person who assists the mother during childbirth and initially acquired her skills by delivering babies herself or through apprenticeship to other traditional birth attendants.” ; Skilled health personnel: “an accredited health professional - such as a midwife, doctor or nurse - who has been educated and trained to proficiency in the skills needed to manage normal (uncomplicated) pregnancies, childbirth and the immediate postnatal period, and in the identification, management and referral of complications in women and newborns.” .

Because definitions of TBA and skilled health personnel have varied over time , we will also consider alternative definitions. Other participants are the individuals, families and communities involved in each intervention.

*Examples of types of interventions*

We will only include studies in settings where TBAs were providers of childbirth care, and where an intervention or combination of interventions to increase birth with skilled health personnel was implemented. Examples of types of interventions are listed here, but others will be included if planned or implemented by countries.

**-** interventions to address financial barriers to use skilled health personnel;

**-** interventions to improve access to services by removing geographical and/or financial barriers;

**-** interventions to regulate midwifery;

**-** interventions to mobilize the community in favour of delivering with skilled health personnel;

- interventions to provide incentives for institutional childbirth;

**-** interventions concerned with human resources development and/or deployment;

**-** interventions to find new roles for TBAs, including role as a companion at birth; advocacy; teaming approaches which partner a TBA with a midwife or with the health centre, etc.;

- interventions to improve quality of existing services; and

- interventions to improve governance of the health sector (intersectoral support and legislation).

We will include low, middle and high-income countries.

References will be excluded where they only contain these elements:

- there was no specific mention of TBAs attending births;
- TBAs continued to attend births and referred women to skilled health personnel or a facility for complications;
- the intervention was to train TBAs to upgrade their skills to attend birth, or where they were supplied with delivery kits to continue to provide childbirth care;
- barriers to use of care with skilled health personnel or women's preferences for care were described but no intervention was examined; and
- full-text is not available in English, French, Spanish or Portuguese.

*Examples of types of outcomes measures*

As before, we list some examples of outcomes, but others related to these will be included if reported in the included studies.

1) Use of skilled health personnel or health facility:

- use of antenatal care;

- use of postpartum care;

- use of postnatal care;

- % of births in a health facility;

- % of births attended by skilled health personnel; and

- maternal satisfaction with health facilities or skilled health personnel for childbirth services.

2) Measure of maternal mortality:

- maternal deaths and

- maternal mortality ratio.

3) TBA activities:

- TBA advises use of health facilities for childbirth services;

- TBA accompanies women to health centre;

- TBA as companion at birth; and

- % of births with TBAs.

Search methods for identification of studies

*Electronic databases*

African Index Medicus (AIM)

# African Journal Online (AJO)

**African Women's Bibliographic Database**

Bioline International

Cochrane Library

Conference papers index (CSA ILLUMINA)

Cumulative Index to Nursing and Allied Health Literature (CINAHL)

Dissertations and theses (ProQuest)

Eastern Mediterranean Regional Office (EMRO)

Embase

[FRANCIS](http://firstsearch.oclc.org.ezp-prod1.hul.harvard.edu/WebZ/LogDbChange?next=html/advanced.html:dbchoice=1:sessionid=fsapp3-53333-g6mbh8k6-zepr59:entitypagenum=23:0:dbname=FRANCIS)

Health Research and Development Information Network (Herdin)

# IndMED

# Institute of Tropical Medicine (ITM) in Antwerp Belgium

La bibliothèque de Santé Tropicale

Latin American and Caribbean Health Sciences (LILACS)

l'Ecole nationale de la santé publique

Maternity and Infant Care (Ovid)

PAIS International

Population Information Online (POPLINE)

Pubmed

# Royal Tropical Institute (KIT)

Sociofile database (CSA ILLUMINA)

Western Pacific Region Index Medicus (WPRIM)

Women's resources international

World Health Organization library database (WHOLIS)

*Other references sources*

We will contact relevant agencies, discussion forums and experts in the field for information and collection of literature.

*Search strategy*

Pubmed:

"traditional birth attendant"[All Fields] OR "traditional birth attendants"[All Fields] OR "traditional midwife"[All Fields] OR "traditional midwives"[All Fields] OR "traditional midwifery"[All Fields] OR "lay midwife"[All Fields] OR "lay midwives"[All Fields] OR "lay midwifery"[All Fields] OR "traditional family birth attendant" OR "traditional family birth attendants" OR "traditional home birth" OR "family birth" OR "home birth*"[All Fields] OR "homebirth*"[All Fields] OR "home delivery"[All Fields] OR "home deliveries"[All Fields] OR "Home Childbirth"[Mesh] OR "home childbirth*"[All Fields]

Embase:

"traditional birth attendant" OR "traditional birth attendants" OR "home delivery" OR "home deliveries"

Extra terms in French:

1. accoucheuses traditionnelles;

2. accoucheuse traditionnelle;

3. accouchement à domicile.

No extra terms in Spanish and Portuguese will be needed because the selected databases that retrieve documents in these languages allow the search to be performed in English.

The selected databases listed above will be searched using the search strategy modified for each database as necessary.

Data collection and analysis

*References selection and eligibility*

One review author will perform the search and the analysis of the titles and abstracts acquired from all the above cited sources will be reviewed independently by two review authors that will apply the eligibility criteria - one author will review all references while another will independently review 50%. Any disagreement will be resolved by discussion.

Reference Manager for Windows will be used to manage the database.

This systematic review will have two components according to the type and quality of data of the references retrieved:

- references with primary or secondary data analysis assessed of moderate or high quality will be included for analysis;

- references related to the objectives and that fulfil the inclusion criteria, but that do not contain empirical data or analysis of relevant data; that cover extended time periods with multiple and changing interventions but where it is not possible to attribute the reported outcomes to specific interventions; or do not meet our quality criteria, will be kept for reference in a comprehensive inventory, and classified according to country and intervention type.

*Data extraction of the selected references*

Data will be extracted using a pre-designed data recording table (see below) based on the criteria proposed by the Strengthening the Reporting of Observational Studies in Epidemiology (STROBE) statement , the Transparent Reporting of Evaluations with Non-randomized Designs (TREND) statement and the National Public Health Partnership . The key information includes: country, study aims, design, context/intervention description, outcomes/results (only the outcomes/results related to antenatal care, childbirth and postnatal care will be extracted). One author will perform data extraction; the tables will be reviewed and discussed between all authors prior to proceeding.

| **Reference** | **Country** | **Study aims** | **Design** | **Context/ Intervention description** | **Outcomes/results** |
| --- | --- | --- | --- | --- | --- |
|  |  |  |  |  |  |

*Quality appraisal of the selected references*

To determine the quality of the studies for inclusion in the systematic review, two different assessment tools will be used: for qualitative methods, the Critical Appraisal Skills Programme (CASP) assessment tool , and for quantitative methods the McMaster University Occupational Therapy Evidence-Based Practice Research Group quality assessment tool . For mixed methods studies, both tools will be applied. We will appraise each study as high, moderate or low quality, defined as follows: High: quality of the research design and findings reported allow confidence in measures of internal and external validity; Moderate: quality of the research design and findings reported in the publication correspond to some quality assessment criteria, allowing for some level of confidence in the outcomes described, additional information on aspects of the research process would increase confidence; Low: quality of the research design and findings reported correspond to few quality assessment criteria and details allowing for confidence in the outcomes described are lacking.

Review authors will independently appraise the quality of the selected references and data will be compared and any errors and disagreements resolved by discussion.

**References**

1. Skilled care includes the skilled health personnel supported by the functioning health system. [↑](#footnote-ref-2)
